# Population uptake and effectiveness of test‐and‐treat antiretroviral therapy guidelines for preventing the global spread of HIV: an ecological cross‐national analysis

**DOI:** 10.1111/hiv.12750

**Published:** 2019-05-29

**Authors:** A Mendez‐Lopez, M McKee, D Stuckler, R Granich, S Gupta, T Noori, JC Semenza

**Affiliations:** ^1^ Green Templeton College University of Oxford Oxford UK; ^2^ Department of Public Health & Policy London School of Hygiene & Tropical Medicine London UK; ^3^ Dondena Research Centre University of Bocconi Milan Italy; ^4^ Independent Public Health Consultant San Francisco CA USA; ^5^ Independent Public Health Consultant Delhi India; ^6^ European Centre for Disease Prevention and Control Stockholm Sweden

**Keywords:** ecological, health systems, HIV care continuum, structural drivers, test‐and‐treat

## Abstract

**Objectives:**

Although the benefits of adopting test‐and‐treat antiretroviral therapy (ART) guidelines that recommend initiation of ART regardless of CD4 cell counts have been demonstrated at the individual level, there is uncertainty about how this translates to the population level. Here, we explored whether adopting ART guidelines recommending earlier treatment initiation improves population ART access and viral suppression and reduces overall disease transmission.

**Methods:**

Data on ART initiation guidelines and treatment coverage, viral suppression, and HIV incidence from 37 European and Central Asian countries were collected from the European Centre for Disease Prevention and Control and the Global HIV Policy Watch and HIV 90‐90‐90 Watch databases. We used multivariate linear regression models to quantify the association of ART initiation guidelines with population ART access, viral suppression, and HIV incidence, adjusting for potential confounding factors.

**Results:**

Test‐and‐treat policies were associated with 15.2 percentage points (pp) [95% confidence interval (CI) 0.8–29.6 pp; *P* = 0.039] greater treatment coverage (proportion of HIV‐positive people on ART) compared with countries with ART initiation at CD4 cell counts ≤ 350 cells/μL. The presence of test‐and‐treat policies was associated with 15.8 pp (95% CI 2.4–29.1 pp; *P* = 0.023) higher viral suppression rates (people on ART virally suppressed) compared with countries with treatment initiation at CD4 counts ≤ 350 cells/μL. ART initiation at CD4 counts ≤ 500 cells/μL did not significantly improve ART coverage compared to initiation at CD4 counts ≤ 350 cells/μL but achieved similar degrees of viral suppression as test‐and‐treat.

**Conclusions:**

Test‐and‐treat was found to be associated with substantial improvements in population‐level access to ART and viral suppression, further strengthening evidence that rapid initiation of treatment will help curb the spread of HIV.

## Introduction

In 2015, the World Health Organization (WHO) and the European AIDS Clinical Society called for universal test‐and‐treat programmes, with initiation of antiretroviral therapy (ART) immediately upon diagnosis of HIV infection, as a means to reduce rates of HIV‐related illness and mortality and onward transmission 1, 2, 3. The rationale for reducing onward transmission derived primarily from evidence that early treatment reduced the risk of mother‐to‐child transmission and in serodiscordant couples 4, 5, 6, 7, 8, 9, 10, 11, 12, 13, 14, 15, 16. The HIV Prevention Trials Network (HPTN052) trial had found that ART initiation at CD4 counts of between 350 and 550 cells/μL led to a reduction of 96% in HIV transmission compared to delaying ART initiation until the CD4 count was ≤ 250 cells/μL 12, 16. This was consistent with earlier observational studies and supported by systematic reviews 11, 13, 14, 17. Yet, the argument that this would lead to population‐level benefits was controversial. Some argued that expanding ART might create a false sense of security among those affected, perversely encouraging greater rates of unsafe sex 18, 19, 20, which has been contested 21, 22, 23, 24. Others highlighted constraints to scaling up treatment as a result of limited resources, especially in low‐income settings 25, 26, 27, uncertainty about the use of data from clinical trials that showed ‘modest benefits’ 28, nonreplicability at the community level 29, 30, and the risk of increasing rates of adverse effects caused by ART and resistance 26, 31.

In Europe, a key argument centred on whether findings in couples could be generalized to the wider population, especially as the incidence was lower than in other parts of the world and, in many European countries, was declining. This reflected the limited evidence at the population level, with studies producing mixed findings but often suggesting that population‐level benefits may be more modest than those found in trials at the individual level. A number of ecological studies have been carried out, but mostly in single communities. An association between greater ART coverage and lower viral loads and transmission has been reported in diverse settings, including British Columbia in Canada 32, 33, 34, San Francisco in the USA 35, KwaZulu‐Natal in South Africa 17, 36, and Taiwan 37. One cross‐national study found that expanding ART coverage in the 30 highest AIDS mortality burden countries correlated with reduced mortality rates from HIV‐related causes 38. However, a recent review argued that findings from existing population‐level studies were mixed, with one study reporting decreasing risk per contact among those on ART being counteracted by more unsafe sexual episodes 18. Another review found that test‐and‐treat appeared to be less effective at the population level than anticipated from modelling studies 29, 30.

**Figure 1:**
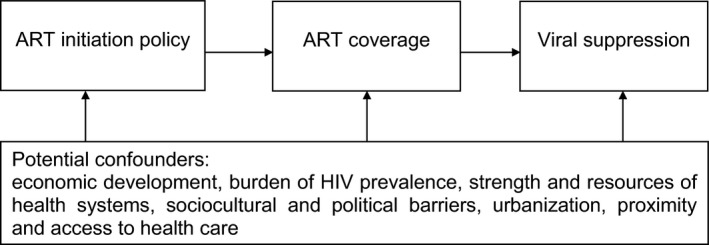
Conceptual framework of the relationship between antiretroviral therapy (ART) initiation policies, ART coverage and viral suppression.

Here, we take advantage of a unique opportunity to test the impact of the expansion of test‐and‐treat policies in 37 European and Central Asian countries. Several countries pre‐empted the adoption of the test‐and‐treat guidelines in WHO's 2015 recommendations, while others have yet to change (see Table S1). These marked differences in timing enabled us to test the hypothesis that expanding test‐and‐treat guidelines increases population access to ART coverage and, in so doing, improves viral suppression and reduces HIV incidence (as described in Fig. 1).

## Methods

### Data sources

We collected data on the prevalence of HIV‐positive status, the proportion of people diagnosed with HIV infection with access to ART, and proportion of people on ART with viral suppression from the European Centre for Disease Prevention and Control (ECDC)'s *HIV Treatment and Care* and *Continuum of HIV Care* reports covering 37 countries in the WHO European Region 39, 40. This includes the European Union and European Economic Area (EU/EEA) (24 countries) as well as Eastern European and Central Asian countries (13 countries). ECDC's system for monitoring progress against the Dublin Declaration on Partnership to Fight HIV/AIDS has been described elsewhere 39, 40, 41, 42; briefly, ECDC surveys health ministries and other health authorities for data on access to ART and viral suppression. The years for which data are available vary across countries between 2012 and 2016, with most (26 countries) providing data for the year 2015. Table S2 lists all 37 countries included in the analyses and details the year of available data for each country. The data sources also varied across countries 39. For instance, for the number of people with HIV infection who are on ART, 29% of countries used surveillance data, 26% used cohort data, and 45% used another data source. For reporting the number of people who are virally suppressed, 26% of countries used surveillance data, 44% used cohort data, and 30% used another data source. Table S3 provides detailed information of the data source used in each country.

The European Centre for Disease Prevention and Control has produced definitions to harmonize reporting practices 41. Current ART status is based on persons using ART, irrespective of treatment regimen or treatment interruptions and discontinuations. Viral suppression is defined as having initiated treatment and achieved a viral load ≤ 200 HIV‐1 RNA copies/mL of blood at the last attendance for HIV care. Nonetheless, countries employed slightly varying definitions for reporting data on ART prevalence and viral suppression, introducing measurement errors 41, 42, 43. Data on access to ART and viral suppression in Kazakhstan and Kyrgyzstan only included patients who were ≥ 15 years old.

Data on new HIV infection rates per 100 000 population were obtained for all countries from The European Surveillance System (TESSy) to which countries provide surveillance data, as reported by ECDC and the WHO Regional Office for Europe 44. To capture changes in HIV transmission, we calculated the growth rate as the difference between the rate in the year for which each country had available data on the other variables (ART access and viral suppression) and the rate in the previous year. However, these data have limits in capturing reduced HIV transmission, as newly reported HIV diagnoses include recently infected individuals as well as those who were infected several years ago but only recently tested for HIV 44.

Data on ART guidelines in the year for which the data were available were taken from the ECDC Dublin Declaration monitoring and country reports 40, 45, 46, the Global HIV Policy Watch database (June 2017 edition) 47, and the HIV 90‐90‐90 Watch database (May 2017 edition) 48. Table S1 summarizes the ART policies that countries had in place in the year for which the HIV data were available. Where discrepancies occurred, we used the more comprehensive HIV Policy Watch database. Guidelines were categorized into three groups: those recommending ART initiation at CD4 cell counts ≤ 350 cells/μL, ART initiation at CD4 counts ≤ 500 cells/μL, and universal treatment (i.e. test‐and‐treat or treatment initiation irrespective of CD4 count). No country in the sample used the 2003 WHO HIV treatment guidelines recommending treatment initiation at CD4 counts ≤ 200 cells/μL.

### Statistical modelling

To adjust for potential confounding factors, we used multivariate linear regression models, corresponding to the causal chain outlined in Figure 1:

$$\text{Prevalence of HIV‐positive persons with ART}\;{\text{access}}_{i}=\alpha \;+\;\beta_{1}\;\text{ART}\;{\text{guidelines}}_{i}\;+\;\beta_{2}\;\text{log}\;{\text{GDP}}_{i}\;+\;\beta_{3}\;\text{public health}\;{\text{expenditure}}_{i}\;+\;\beta_{4}\;\text{HIV}\;{\text{prevalence}}_{i}\;+\;\beta_{5}\;\text{region}\;+\;\varepsilon_{i}$$

Here, *i* is country, GDP is gross domestic product per capita and ε is the error term. ART guideline is coded as an ordinal variable, as described above. Log GDP per capita is in international constant 2011 purchasing power parity‐adjusted US dollars to facilitate cross‐national comparisons and adjust for positive skew. To address the possibility that wealthier nations may achieve greater access, we also adjusted for public health expenditures per capita in international constant 2011 US dollars adjusted for purchasing power parity and inflation. We also included additional adjustments for country HIV burden and region (EU/EEA or Eastern Europe and Central Asia). Data on the number of people with HIV infection were taken from ECDC 39 and data on the total population were taken from the World Bank World Development Indicators (WDI) database to calculate HIV prevalence. All other data on control variables were taken from the World Bank WDI database for the year 2015, corresponding to the year in which most countries reported HIV data, except for public health expenditures, which were from 2014, the latest year in which all countries provided data 49.

In the second step, we quantified the association between ART initiation policy and viral suppression, performing a mediation analysis, as follows:

$$\text{Prevalence of HIV‐positive persons with viral}\;{\text{suppression}}_{i}=\alpha \;+\;\beta_{1}\;\text{ART}\;{\text{guidelines}}_{i}\;+\;\beta_{2}\;\text{log}\;{\text{GDP}}_{i}\;+\;\beta_{3}\;\text{public health}\;{\text{expenditure}}_{i}\;+\;\beta_{4}\;\text{HIV}\;{\text{prevalence}}_{i}\;+\;\beta_{5}\;\text{region}\;+\;\varepsilon_{i}$$

Finally, we investigated the association between ART initiation guidelines and the growth rate of new HIV infections per 100 000 population as a proxy for change in transmissibility:

$$\text{Growth rate of HIV incidence per}\;100\;000_{i}=\alpha \;+\;\beta_{1}\;\text{ART}\;{\text{guidelines}}_{i}\;+\;\beta_{2}\;\text{log}\;{\text{GDP}}_{i}\;+\;\beta_{3}\;\text{public health}\;{\text{expenditure}}_{i}\;+\;\beta_{4}\;\text{HIV}\;{\text{prevalence}}_{i}\;+\;\beta_{5}\;\text{region}\;+\;\varepsilon_{i}$$

To account for potential heteroscedasticity, robust standard errors were used. In view of the small sample size and potential overfitting, we present for both models, first, unadjusted, more parsimonious results, and, secondly, fully specific models adjusted for several controls. All models were estimated using stata, version 13.0 (StataCorp, College Station, TX).

## Results

### Impact of test‐and‐treat guidelines on population‐level ART access among people with diagnosed HIV infection

An estimated 1.2 million people were living with HIV, of whom 0.7 million were receiving ART (< 60%). In the EU/EEA (24 countries), 77.5% of the people diagnosed with HIV infection were on ART and, of those, 86.6% had attained viral suppression. These estimates, respectively, were lower, at 57.1% and 59.8%, in Eastern Europe and Central Asia (13 countries). Table S4 describes the HIV data obtained from the 37 countries in the sample.

At the time the data were published, in April 2017, 14 of the 37 countries had yet to adopt test‐and‐treat guidelines, which corresponds to about 25% of the population in the 37 countries not covered by universal HIV treatment. We observed that seven countries (all from EU/EEA) began test‐and‐treat prior to the WHO 2015 guideline update, corresponding to about 20% of the countries included in the sample and covering 35% of the population in the sample countries. Of the 14 countries that have not updated their guidelines to the latest (2015) WHO and EACS recommendations at the time the data were published in 2017, 11 (29.7% of the countries and about 21% of the total population in the sample countries) had guidelines recommending treatment at CD4 cell counts ≤ 500 cells/μL, corresponding to the WHO guidelines of 2013. The guidelines of three countries (Azerbaijan, Lithuania and Tajikistan; 8.1% of the countries and 4% of the sample population) recommend initiation of ART at CD4 cell counts ≤ 350 cells/μL, corresponding to the 2010 WHO guidelines. Of the 14 countries that have not adopted test‐and‐treat, four countries are from the EU/EEA: Belgium, Bulgaria and Luxemburg (CD4 count ≤ 500 cells/μL), and Lithuania (CD4 count ≤ 350 cells/μL). Table S3 summarizes the state of the ART policies for the studied countries.

**Figure 2:**
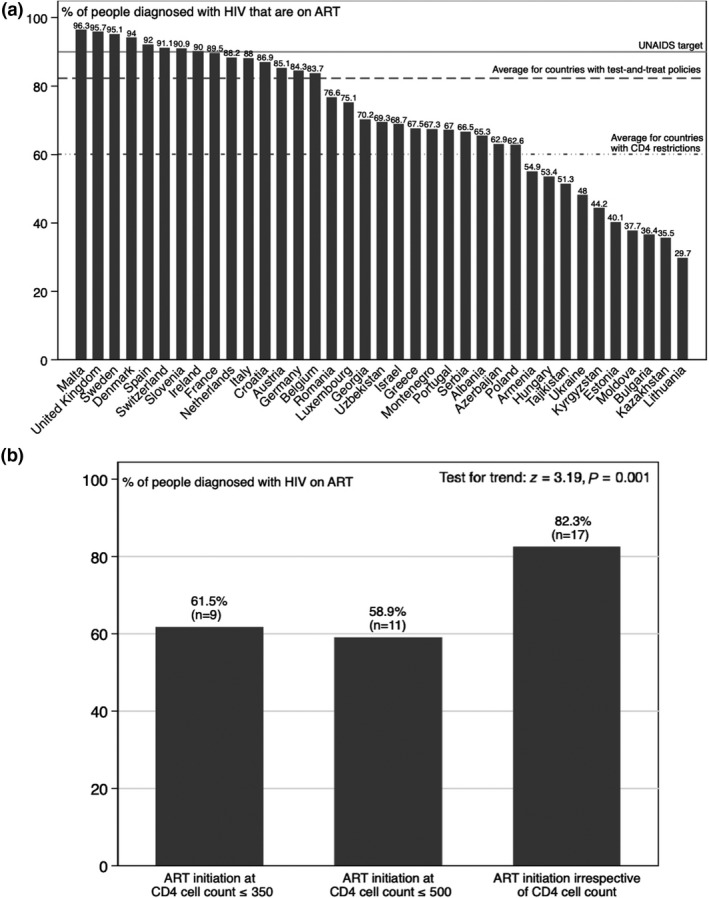
Population‐level access to antiretroviral therapy (ART) and ART initiation guidelines. (a) Percentage of people diagnosed with HIV infection on ART by country and average percentage by ART initiation policy. (b) Percentage of people diagnosed with HIV infection on ART by ART initiation policy.

Figure 2a (panel 1) shows the proportion of people on ART out of all people living with diagnosed HIV infection by country. Unadjusted, test‐and‐treat policies achieve greater access to ART (mean 82.4%), compared with policies that employ CD4 threshold restrictions (mean 60.1%). Countries with test‐and‐treat policies had, on average, 22.3 percentage points more diagnosed HIV‐positive people on ART (two‐tailed *t*‐test: *t* = 4.03; *P* = 0.0003). As shown in Figure 2b, an increase in access to ART appeared to occur most often when moving from any CD4 restriction to test‐and‐treat, rather than from the threshold of CD4 count ≤ 350 cells/μL to CD4 count ≤ 500 cells/μL. Table S2 describes the ART initiation guidelines in the year data were available in each of the countries shown in Figure 2a.

Table 1 shows the results of our multivariate linear regression model quantifying the association of test‐and‐treat with population‐level access to ART among people with diagnosed HIV infection. Countries that had test‐and‐treat policies had a 20.8 (95% CI 5.2–36.4; *P* = 0.01) percentage points increase of people on ART compared with countries with guidelines recommending treatment initiation at CD4 counts ≤ 350 cells/μL. After controlling for the level of economic development, HIV prevalence, subregion and public health care expenditure, the estimated effect size was attenuated to 15.2 percentage points (95% CI 0.8–29.6; *P* = 0.039), appearing to reflect mainly how better resourced and wealthier health systems also achieved higher rates of uptake.

**Table 1:** Association of antiretroviral therapy (ART) initiation guidelines with population‐level access to ART among people diagnosed with HIV infection

	Percentage of people diagnosed with HIV infection on ART	
	Model 1	Model 2
ART initiation guidelines		
Initiation at CD4 count ≤ 350 cells/μL	Reference	Reference
Initiation at CD4 count ≤ 500 cells/μL	−2.65 (−19.1 to 13.8)	0.23 (−13.3 to 13.8)
Initiation at any CD4 count (test‐and‐treat)	20.8* (5.20–36.4)	15.2* (0.82–29.6)
Per 1% increase in GDP per capita (≈$927)		1.58 (−7.89 to 11.1)
Per $1000 increase in health care expenditure per capita		6.55* (1.61–11.5)
Per 1 SD increase in HIV prevalence (SD = 0.17%)		−4.77 (−10.6 to 1.03)
Region		
EU/EEA		Reference
Eastern Europe and Central Asia		3.04 (−14.7 to 20.8)
Number of countries	37	37
*R* ^2^	0.319	0.626

A constant was included in all models but is not shown. 95% confidence intervals are shown in brackets. Model 1: unadjusted; model 2: adjusted for level of economic development, HIV prevalence, subregion and public health care expenditure.

EU/EEA, European Union and European Economic Area; SD, standard deviation.

*P <* *0.05.

### Impact of test‐and‐treat on population‐level viral suppression among people on ART

**Figure 3:**
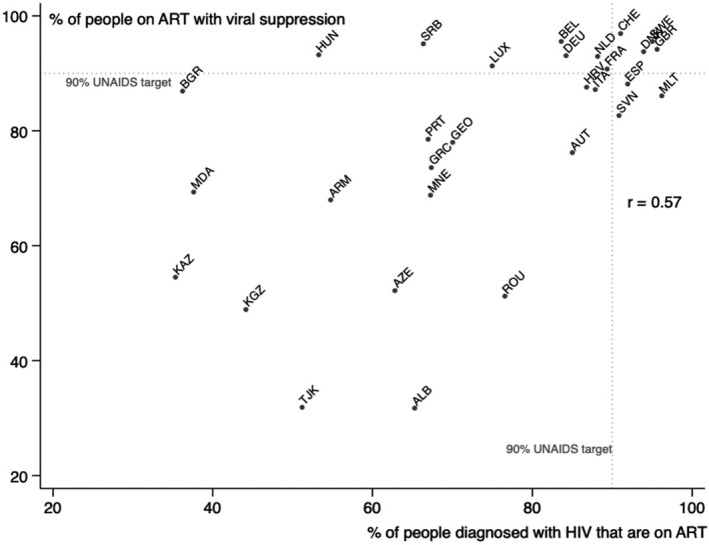
Association between the percentage of people diagnosed with HIV infection on antiretroviral therapy (ART) and the percentage of people on ART with viral suppression.

Figure 3 depicts the positive unadjusted association between the percentage of people diagnosed with HIV infection who were on ART and the percentage of people diagnosed with HIV who were on ART with viral suppression (*r* = 0.57; *P* = 0.001). It shows that countries with higher ART coverage were more likely to achieve a higher percentage of population‐level viral suppression among people on ART.

**Figure 4:**
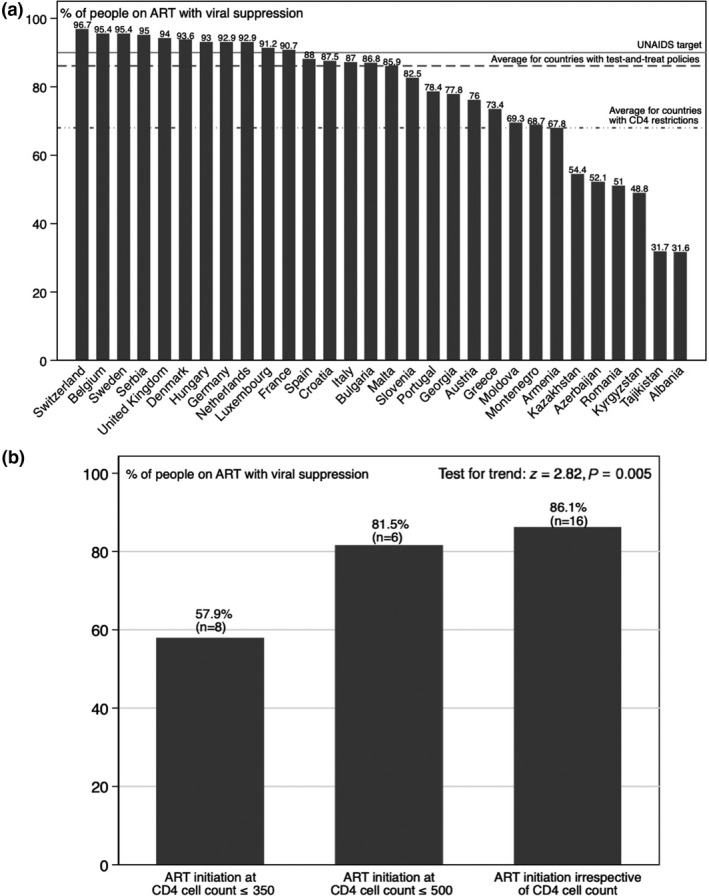
Population‐level viral suppression and antiretroviral therapy (ART) initiation guidelines. (a) Percentage of people on ART with viral suppression by country and average percentage by ART initiation policy. (b) Percentage of people on ART with viral suppression by ART initiation policy.

As shown in the unadjusted plots in Figure 4a (panel 2), we observed that test‐and‐treat policies were associated with a higher proportion of population‐level viral suppression among HIV‐positive patients on ART (mean 86.1%) compared with countries with CD4 restrictions for ART initiation (mean 67.9%). Countries with ART policies offering treatment for all had, on average, 18.1 percentage points more viral suppression among patients on ART compared with countries restricting treatment only to patients with low CD4 cell counts; this difference was significant at the 5% level (two‐sample *t*‐test: *t* = 2.91; *P* < 0.007). Figure 4b shows a steady increase in the average percentage of people on ART with viral suppression as the inclusiveness of the three ART initiation policies increased, whereby, in countries with the most inclusive treatment guidelines, i.e. test‐and‐treat, the populations achieved the highest levels of viral suppression.

After adjusting for potential confounders in a multivariate linear regression model, as shown in Table 2, we found that test‐and‐treat was associated with 15.8 percentage points more of people on ART achieving viral suppression compared with countries with treatment initiation at CD4 counts ≤ 350 cells/μL (95% CI 2.4–29.1%; *P* = 0.007). Countries with guidelines recommending initiation of ART at CD4 cell counts ≤ 500 cells/μL achieved similar levels of viral suppression to countries with test‐and‐treat policies (15.1 percentage points; 95% CI 1.2–29.1; *P* = 0.023).

**Table 2:** Association of antiretroviral therapy (ART) initiation guidelines with population‐level viral suppression among people on ART

	Percentage of people on ART with viral suppression	
	Model 1	Model 2
ART initiation guidelines		
Initiation at CD4 count ≤ 350 cells/μL	Reference	Reference
Initiation at CD4 count ≤ 500 cells/μL	17.6 (−4.06 to 39.3)	15.1* (1.18–29.0)
Initiation at any CD4 count (test‐and‐treat)	26.9** (7.99–45.9)	15.8* (2.39–29.1)
Per 1% increase in GDP per capita (≈$927)		9.95* (0.71–19.2)
Per $1000 increase in health care expenditure per capita		1.66 (−2.79 to 6.12)
Per 1 SD increase in HIV prevalence (SD = 0.17%)		0.20 (−4.99 to 5.38)
Region		
EU/EEA		Reference
Eastern Europe and Central Asia		−2.71 (−21.4 to 16.0)
Number of countries	30	30
*R* ^2^	0.335	0.648

A constant was included in all models but is not shown. 95% confidence intervals are shown in brackets. Model 1: unadjusted; model 2: adjusted for level of economic development, HIV prevalence, subregion and public health care expenditure.

EU/EEA, European Union and European Economic Area; SD, standard deviation.

*P <* *0.05; **P <* *0.01.

### Impact of test‐and‐treat on HIV transmission

Table 3 shows the results of our multivariate linear regression model quantifying the association of ART initiation guidelines and the growth rate of new HIV infections per 100 000 population. We found that, after adjusting for potential confounders, countries with ART initiation at CD4 cell counts ≤ 500 cells/μL had an associated 16% reduction in their new HIV infection rates per 100 000, compared with countries with ART initiation at CD4 cell counts ≤ 350 cells/μL (95% CI −30 to −1.4%; *P* = 0.033). No difference was observed between countries with test‐and‐treat ART guidelines and countries with ART initiation at CD4 cell counts ≤ 350 cells/μL (−6.3%; 95% CI −23.5 to 10.9%; *P* = 0.46).

**Table 3:** Association of antiretroviral therapy (ART) initiation guidelines with the growth rate of new HIV infections per 100 000 population

	Per cent change in new HIV infection rate per 100 000	
	Model 1	Model 2
ART initiation guidelines		
Initiation at CD4 count ≤ 350 cells/μL	Reference	Reference
Initiation at CD4 count ≤ 500 cells/μL	−17* (−0.30 to −0.043)	−16* (−0.30 to −0.014)
Initiation at any CD4 count (test‐and‐treat)	−8.2 (−0.21 to 0.043)	−6.3 (−0.24 to 0.11)
Per 1% increase in GDP per capita (≈$927)		−0.031 (−0.095 to 0.033)
Per $1000 increase in health care expenditure per capita		−0.00054 (−0.044 to 0.043)
Per 1 SD increase in HIV prevalence (SD = 0.17%)		−0.025 (−0.053 to 0.0036)
Region		
EU/EEA		Reference
Eastern Europe and Central Asia		−0.013 (−0.18 to 0.16)
Number of countries	36	36
*R* ^2^	0.217	0.262

A constant was included in all models but is not shown. 95% confidence intervals are shown in brackets. Model 1: unadjusted; model 2: adjusted for level of economic development, HIV prevalence, subregion and public health care expenditure.

EU/EEA, European Union and European Economic Area; SD, standard deviation.

*P <* *0.05.

### Robustness check

As country‐years of data availability varied, we also included a variable for the year of data availability to adjust for the potential effect of secular trends. None of the results was qualitatively unchanged.

## Discussion

We found a significant association between adoption of test‐and‐treat guidelines and greater access to both ART and viral suppression compared with the use of a CD4 count ≤ 350 cells/μL threshold, even after adjusting for potential confounding factors. While we found that test‐and‐treat was associated with greater access to ART when compared with the most restrictive ART initiation policy (CD4 counts ≤ 350 cells/μL), there were no statistically significant differences between ART initiation at CD4 counts ≤ 500 cells/μL and at CD4 counts ≤ 350 cells/μL. This indicates that the effect of test‐and‐treat on expanding ART coverage is probably substantial in comparison with any alternative ART initiation policy. We also found that greater viral suppression was achieved with both test‐and‐treat and ART initiation at CD4 counts ≤ 500 cells/μL compared with ART initiation at CD4 counts ≤ 350 cells/μL, which is important given that this is associated with reduced HIV‐related illness, deaths, and transmissibility 35, 50. Community viral load has been proposed as an effective population‐level biomarker of HIV burden and as a novel means of assessing the potential impact of population‐level HIV prevention and treatment interventions 50. When we studied the association between ART initiation policy and per cent change in new HIV infection rates, we observed that earlier treatment initiation at CD4 counts ≤ 500 cells/μL was associated with a decline in new HIV infections but initiation irrespective of CD4 cell count was not associated with a decline when compared with initiation at CD4 counts ≤ 350 cells/μL.

Our findings are consistent with those of other studies. First, the association between test‐and‐treat and higher ART coverage is both intuitive and consistent with the findings of other studies showing earlier initiation of ART to be associated with increased ART coverage 51, 52. Secondly, the association between test‐and‐treat and greater population‐level viral suppression among people on ART is also consistent with the results of several studies that reported that increased ART coverage and earlier initiation of ART were linked to improved HIV outcomes 32, 33, 34, 36, 37, 38, 52, 53, 54, 55, including attainment of viral suppression 35, 50, 56, 57 and reduced transmission and incidence 35, 50. Greater viral suppression of the population under treatment initiation guidelines that recommend therapy for all and for those with higher CD4 cell counts (CD4 counts ≤ 500 cells/μL) might be explained by the steady access to ART that confer these more inclusive policies offering treatment to a greater proportion of the HIV‐positive population in contrast to an ART initiation policy that delays treatment initiation until patients reach lower CD4 cell counts.

The apparent effectiveness of test‐and‐treat in increasing population‐level ART coverage among people with diagnosed HIV infection and increasing viral suppression among people on ART reinforces the decision by some countries, beginning in 2011, to adopt ART irrespective of CD4 cell count and immediate initiation of ART with simplified regimens; and it also reinforces the recommendations by the WHO and EACS in 2015 to adopt test‐and‐treat policies as a contribution to achieving the UNAIDS 90‐90‐90 target. However, this will only be possible with progress in the first stage in the HIV care cascade, the 90% diagnosis target, as even a small number of undiagnosed people could sustain an epidemic 58. Also, other stages of the HIV care continuum not directly measured in the 90‐90‐90 scheme are important to reach the UNAIDS target, such as linking diagnosed HIV‐positive patients to services and ensuring sustained and appropriate care, like switching to second‐line therapy regimens when necessary 27. Indeed, we found increased access to ART and viral suppression in stronger health systems, which are likely to provide better retention within the system because of increased availability, accessibility and affordability of services. Thus, achieving the 90‐90‐90 target will require both HIV‐specific measures, such as test‐and‐treat policies and universal access to ART, and also general improvements to the health system.

There are some limitations to this study. The first is the risk of ecological fallacy. While exposure to a given ART initiation guideline is a national policy that should apply to everyone, we cannot exclude the possibility that some populations subject to stigma and discrimination might be treated differently. For instance, only about half of the countries in Europe and Central Asia offer HIV treatment to undocumented migrants 59. Other populations that are also likely to suffer differential provision of HIV health care services are sex workers 60, 61 and people who inject drugs 62, 63. Consequently, our results are likely to be conservative estimates of the association between ART initiation policies and population‐level access to ART. A second, linked, limitation is the assumption that adoption of the guidelines translated into implementation. If failure to implement guideline recommendations was randomly distributed across various ART initiation policies, this would have yielded conservative estimates. Nonrandom variation would have yielded biased estimates. However, many countries adopted the guidelines some time before the reporting date and can be expected to have rolled them out to ART services. Thirdly, there could be measurement error in the outcome variables as a result of different data collection procedures and misclassification of outcome status, which could have biased our estimates, diluting findings and making it harder to ascertain an association. Indeed, some countries reported very low levels of viral suppression; however, it is possible that these measurements were affected by different factors, including treatment disruptions (voluntary or as a result of stock‐outs), use of first or second line of treatment, loss to follow‐up, transfer of care (people moving to another clinic may show up as having been lost to follow‐up), out‐migration, poor monitoring systems (tracking people and collecting data), and infrequent viral load measurements. A fourth limitation is the small sample size used in the study, potentially generating imprecise estimates of associations. However, the finding of similar results in the larger sample, notwithstanding the greater data problems, offers reassurance. A fifth limitation is that, of the 48 countries that responded to the survey, 37 countries had data available on ART coverage and 30 had data on viral suppression of all people living with HIV 41, 42. Those that either failed to respond or lacked data were mainly from non‐EU/EEA, Eastern European and Central Asian countries that might have different characteristics from the countries included in the analyses, which could have biased our estimates. Sixthly, we used cross‐sectional data measuring exposure and outcome simultaneously. Reverse causality is a risk but is unlikely because, while it is theoretically possible that a greater proportion of people on ART and virally suppressed could have exerted pressure for expanded ART initiation policies, the association is much more likely to flow from expanded ART initiation policies to a greater percentage of people on ART and virally suppressed. Another limitation of cross‐sectional data is that they do not capture any lag effect between policy adoption and treatment initiation. If there were lagged effects, our estimates could be biased towards the null. Finally, the country data used were drawn from different years, reflecting the limitations of the country surveillance and reporting systems; however, this did not affect our ability to test our question across countries and years, and as a robustness check the models were adjusted for the year of data availability to account for the potential effect of secular trends.

To our knowledge, this is the first ecological cross‐national analysis evaluating the impact of test‐and‐treat on achievement of the 90‐90‐90 target internationally, adjusting for several possible confounders. We offer evidence of an association between test‐and‐treat guidelines and greater treatment coverage and viral suppression rates at the population level among people with diagnosed HIV infection and on ART, respectively. While it cannot be regarded as conclusive, it provides evidence that must be assessed further using other research designs, which could, for instance, include longitudinal data for all countries. The cross‐national character of this study, including a broad variety of countries from Europe and Central Asia, gives external validity to the findings and the potential to generalize to other contexts.

Our results have important policy implications. First, they support the recommendation of test‐and‐treat policies by the WHO and EACS as a means to achieve the UNAIDS 90‐90‐90 target for 2020, which also align with the Sustainable Development Goal target to end the HIV epidemic by 2030. Secondly, they show that test‐and‐treat policies can achieve results comparable to those that might otherwise be expected with very large investments in public health services. Thirdly, they reinforce the case for intensification of efforts to expand new test‐and‐treat policies in Eastern European and Central Asian countries. However, our results do not provide guidance on how to implement test‐and‐treat. A next step in research would be to test empirically how to operationalize its implementation, along with the necessary services. Taken together, our results are consistent with a growing body of research indicating that test‐and‐treat is an effective means to reducing the burden of disease attributable to HIV.

## Author contributions

JCS initiated the study. AML and DS designed the study. AML collected the data, implemented the study, and wrote the first draft of the manuscript. MM, DS, RG, SG, TN and JCS offered comments on the draft and helped interpret the findings.

## Supporting information


**Table S1.** Latest antiretroviral therapy initiation guidelines by country and year of adoption for countries with test‐and‐treat (as of August 2017).
**Table S2.** List of countries, region, year of data availability, and antiretroviral therapy policy in the year of data availability.
**Table S3.** Summary of ECDC data sources for 90‐90‐90: access to ART and viral suppression.
**Table S4.** Descriptive statistics for countries.
