# Supplementary material for: Population uptake and effectiveness of test‐and‐treat antiretroviral therapy guidelines for preventing the global spread of HIV: an ecological cross‐national analysis
Source: HIV Med. 2019 May 29;20(8):501–12. doi: 10.1111/hiv.12750 (PMC6772052; doi:10.1111/hiv.12750)
Supplement: Supplementary file 1 — Table S1. Latest antiretroviral therapy initiation guidelines by country and year of adoption for countries with test‐and‐treat (as of August 2017). Table S2. List of countries, region, year of data availability, and antiretroviral therapy policy in the year of data availability. Table S3. Summary of ECDC data sources for 90‐90‐90: access to ART and viral suppression. Table S4. Descriptive statistics for countries. [file HIV-20-501-s001.docx]

**ONLINE SUPPLEMENTARY FILE**

Supplementary file A1. Table A1 – Latest antiretroviral therapy initiation guidelines by country and year of adoption for countries with test-and-treat (as of August 2017)

Supplementary file A2. Table A2 – List of countries, region, year of data availability, and antiretroviral therapy policy in the year of data availability

Supplementary file A3. Table A3 – Summary of ECDC Data sources for the 90-90-90: access to ART and viral suppression

Supplementary file A4. Table A4 – Descriptive statistics of countries

**Table A1** – Latest antiretroviral therapy initiation guidelines by country and year of adoption for countries with test-and-treat (as of August 2017)

| **Country** | **Region** | **CD4 cell count treatment threshold** | | | | **Year adoption test-and-treat** | |
| --- | --- | --- | --- | --- | --- | --- | --- |
|  |  | Initiation regardless of CD4 count | Initiation at CD4 ≤500 | Initiation at CD4 ≤350 |  | |  |
| Albania | Eastern Europe & Central Asia |  | x |  |  | |  |
| Armenia | Eastern Europe & Central Asia |  | x |  |  | |  |
| Austria | EU/EEA | x |  |  | 2014 | |  |
| Azerbaijan | Eastern Europe & Central Asia |  |  | x |  | |  |
| Belgium | EU/EEA |  | x |  |  | |  |
| Bulgaria | EU/EEA |  | x |  |  | |  |
| Croatia | EU/EEA | x |  |  | 2015 | |  |
| Denmark | EU/EEA | x |  |  | 2011 | |  |
| Estonia | EU/EEA | x |  |  | 2016 | |  |
| France | EU/EEA | x |  |  | 2013 | |  |
| Georgia | Eastern Europe & Central Asia | x |  |  | 2015 | |  |
| Germany | EU/EEA | x |  |  | 2015 | |  |
| Greece | EU/EEA | x |  |  | 2016 | |  |
| Hungary | EU/EEA | x |  |  | 2016 | |  |
| Ireland | EU/EEA | x |  |  | 2017 | |  |
| Israel | Eastern Europe & Central Asia |  | x |  |  | |  |
| Italy | EU/EEA | x |  |  | 2011 | |  |
| Kazakhstan | Eastern Europe & Central Asia |  | x |  |  | |  |
| Kyrgyzstan | Eastern Europe & Central Asia |  | x |  |  | |  |
| Lithuania | EU/EEA |  |  | x |  | |  |
| Luxembourg | EU/EEA |  | x |  |  | |  |
| Malta | EU/EEA | x |  |  | 2016 | |  |
| Moldova | Eastern Europe & Central Asia |  | x |  |  | |  |
| Montenegro | Eastern Europe & Central Asia | x |  |  |  | |  |
| Netherlands | EU/EEA | x |  |  | 2012 | |  |
| Poland | EU/EEA | x |  |  | 2016 | |  |
| Portugal | EU/EEA | x |  |  | 2016 | |  |
| Romania | EU/EEA | x |  |  | 2013 | |  |
| Serbia | Eastern Europe & Central Asia | x |  |  | 2017 | |  |
| Slovenia | EU/EEA | x |  |  | 2016 | |  |
| Spain | EU/EEA | x |  |  | 2014 | |  |
| Sweden | EU/EEA | x |  |  | 2014 | |  |
| Switzerland | EU/EEA | x |  |  | 2015 | |  |
| Tajikistan | Eastern Europe & Central Asia |  |  | x |  | |  |
| Ukraine | Eastern Europe & Central Asia |  | x |  |  | |  |
| United Kingdom | EU/EEA | x |  |  | 2015 | |  |
| Uzbekistan | Eastern Europe & Central Asia |  | x |  |  | |  |

**Table A2** – List of countries, region, year of data availability, and antiretroviral therapy policy in the year of data availability

| **Country** | **Region** | **CD4 cell count treatment threshold** | | | **Year of available data** |
| --- | --- | --- | --- | --- | --- |
|  |  | Initiation regardless of CD4 count | Initiation at CD4 ≤500 | Initiation at CD4 ≤350 |  |
| Albania | Eastern Europe & Central Asia |  |  | X | 2015 |
| Armenia | Eastern Europe & Central Asia |  | X |  | 2015 |
| Austria | EU/EEA |  |  | X | 2013 |
| Azerbaijan | Eastern Europe & Central Asia |  |  | X | 2015 |
| Belgium | EU/EEA |  | X |  | 2014 |
| Bulgaria | EU/EEA |  | X |  | 2015 |
| Croatia | EU/EEA | X |  |  | 2015 |
| Denmark | EU/EEA | X |  |  | 2014 |
| Estonia | EU/EEA | X |  |  | 2015 |
| France | EU/EEA | X |  |  | 2013 |
| Georgia | Eastern Europe & Central Asia | X |  |  | 2015 |
| Germany | EU/EEA | X |  |  | 2015 |
| Greece | EU/EEA |  |  | X | 2013 |
| Hungary | EU/EEA | X |  |  | 2015 |
| Ireland | EU/EEA |  |  | X | 2015 |
| Israel | Eastern Europe & Central Asia |  | X |  | 2015 |
| Italy | EU/EEA | X |  |  | 2012 |
| Kazakhstan | Eastern Europe & Central Asia |  |  | X | 2015 |
| Kyrgyzstan | Eastern Europe & Central Asia |  | X |  | 2015 |
| Lithuania | EU/EEA |  |  | X | 2015 |
| Luxembourg | EU/EEA |  | X |  | 2015 |
| Malta | EU/EEA | X |  |  | 2016 |
| Moldova | Eastern Europe & Central Asia |  | X |  | 2015 |
| Montenegro | Eastern Europe & Central Asia | X |  |  | 2015 |
| Netherlands | EU/EEA | X |  |  | 2015 |
| Poland | EU/EEA |  | X |  | 2015 |
| Portugal | EU/EEA |  | X |  | 2014 |
| Romania | EU/EEA | X |  |  | 2014 |
| Serbia | Eastern Europe & Central Asia |  |  | X | 2014 |
| Slovenia | EU/EEA | X |  |  | 2015 |
| Spain | EU/EEA |  | X |  | 2013 |
| Sweden | EU/EEA | X |  |  | 2015 |
| Switzerland | EU/EEA | X |  |  | 2015 |
| Tajikistan | Eastern Europe & Central Asia |  |  | X | 2015 |
| Ukraine | Eastern Europe & Central Asia |  | X |  | 2015 |
| United Kingdom | EU/EEA | X |  |  | 2015 |
| Uzbekistan | Eastern Europe & Central Asia |  | X |  | 2015 |

**Table A3** – Summary of ECDC Data sources for the 90-90-90: access to ART and viral suppression

*Number of people with HIV that are on ART* (1)*:*

Cohort data, 26% of countries (n=8):
Austria, Belgium, Bulgaria, Croatia, Denmark, Luxembourg, Netherlands, Sweden

Surveillance data, 29% of countries (n=9):
Czech Republic, Greece, United Kingdom, Georgia, Kyrgyzstan, Montenegro, Tajikistan, Ukraine, Uzbekistan

Other data source, 45% (n=14):
France, Germany, Ireland, Italy, Malta, Norway, Portugal, Romania, Spain, Azerbaijan, Kazakhstan, Moldova, Serbia, Switzerland

*Number of people virally suppressed* (1)*:*

Cohort data, 44% of countries (n=12): Austria, Belgium, Bulgaria, Croatia, Denmark, Germany, Greece, Italy, Luxembourg, Netherlands, Sweden, Serbia

Surveillance data, 26% of countries (n=7):
Czech Republic, United Kingdom, Armenia, Georgia, Kyrgyzstan, Montenegro, Tajikistan

Other data source, 30% of countries (n=8):
France, Hungary, Malta, Portugal, Romania, Spain, Azerbaijan, Kazakhstan

1. European Centre for Disease Prevention and Control (ECDC). Special report: Continuum of HIV care. Monitoring implementation of the Dublin Declaration on Partnership to Fight HIV/AIDS in Europe and Central Asia: 2017 progress report. Stockholm: European Centre for Disease Prevention and Control; 2017.

**Table A4** – Descriptive statistics of countries

|  | **Countries, N** | **Total number, N (range)** |
| --- | --- | --- |
|  |  |  |
| Estimated number of people living with HIV (PLHIV) | 37 | 1,199,107 (194 to 223,000) |
| People living with HIV diagnosed | 37 | 898,461 (147 to 128,300) |
| People living with HIV on antiretroviral therapy (ART) | 37 | 698,638 (99 to 114,825) |
| People living with HIV with viral suppression | 30 | 524,781 (68 to 104,108) |
|  |  |  |
|  |  | **Mean (range), SD** |
|  |  |  |
| % of PLHIV diagnosed – 1^st^ 90 UNAIDS target | 37 | 72.8% (38.2 to 98.3), 15.6 |
| % of PLHIV diagnosed on ART – 2^nd^ 90 target | 37 | 70.3% (29.7 to 96.3), 19.9 |
| % of PLHIV diagnosed on ART that have viral suppression – 3^rd^ 90 target | 30 | 77.7% (31.6 to 96.8), 19.1 |
|  |  |  |
| HIV prevalence | 37 | 0.17% (0.01% to 0.84%), 0.17 |
| GDP per capita in US dollars, purchasing power parity (PPP) | 37 | $28,407.4 ($2,640.3 to $95,311.1), 18,956.3 |
| Public healthcare expenditures per capita in US dollars, PPP | 37 | $1,792.6 (50.5 to 5,463.6), 1,544.1 |
|  |  |  |
|  |  | **Proportion** |
| ART guidelines CD4 cell count threshold |  |  |
| Initiation at CD4 cell count ≤350 | 11 | 29.7% |
| Initiation at CD4 cell count ≤500 | 10 | 27.1% |
| Initiation regardless of CD4 cell count (test-and-treat) | 16 | 43.2% |
|  |  |  |
| Year of available data for the UNAIDS 90-90-90 target |  |  |
| 2012 | 1 | 2.7% |
| 2013 | 4 | 10.8% |
| 2014 | 5 | 13.5% |
| 2015 | 26 | 70.3% |
| 2016 | 1 | 2.7% |
|  |  |  |
| Region |  |  |
| EU/EEA | 24 | 64.9% |
| Eastern Europe and Central Asia | 13 | 35.1% |
